# An Improved Cole–Cole Model for Characterizing In Vivo Dielectric Properties of Lung Tissue at Different Tide Volumes: An Animal Study

**DOI:** 10.3390/bioengineering12050445

**Published:** 2025-04-24

**Authors:** Yangchun Qin, Liang Zhang, Tixin Han, Yifan Liu, Xuechao Liu, Feng Fu, Hang Wang, Shuoyao Qu, Zhanqi Zhao, Lin Yang, Meng Dai

**Affiliations:** 1 Department of Military Biomedical Engineering, The Fourth Military Medical University, Xi’an 710032, China; yangcq_bme@163.com (Y.Q.); tixinhan@fmmu.edu.cn (T.H.); liuyifan@fmmu.edu.cn (Y.L.); xuechaoliu@fmmu.edu.cn (X.L.); fengfu@fmmu.edu.cn (F.F.); 2 Basic Medical Science Academy, The Fourth Military Medical University, Xi’an 710032, China; zl.bb.ym@163.com; 3 Department of Aerospace Medicine, The Fourth Military Medical University, Xi’an 710032, China; w-h-ang@163.com; 4 Department of Pulmonary and Critical Care Medicine, Xijing Hospital, The Fourth Military Medical University, Xi’an 710032, China; qsy129@fmmu.edu.cn; 5 Innovation Research Institution, Xijing Hospital, The Fourth Military Medical University, Xi’an 710032, China; 6 School of Biomedical Engineering, Guangzhou Medical University, Guangzhou 511436, China; zhanqizhao@gzhmu.edu.cn; 7 Department of Critical Care Medicine, Peking Union Medical College Hospital, Beijing 100730, China

**Keywords:** Cole–Cole model, an improved model, in vivo, dielectric properties of lung tissue, open-ended coaxial probe

## Abstract

Objective: The air content within the lungs directly influences the dielectric properties of lung tissue; however, previous studies were conducted under ex vivo conditions and without quantitatively controlling air volume. This study aims to develop an improved model using in vivo measurements to accurately characterize the dielectric properties of rabbit lung tissue across various tidal volumes. Methods: In this study, six sets of different tidal volumes (30, 40, 50, 60, 70, 80 mL) were set in the frequency band of 100 MHz~1 GHz to analyze the trend of the dielectric properties, and the dielectric parameters were systematically constructed under the conditions of different tidal volumes. Results: It was found that the conductivity and permittivity of rabbit lung tissue showed a decreasing trend with increasing tidal volume in the measuring frequency band. The traditional Cole–Cole model has limitations in simulating the dielectric properties of in vivo lung tissues. Therefore, by refining and optimizing the model, this study successfully reduced the average error between the measured data and the model fitting to less than 5%. Conclusions: This study lays the groundwork for investigating the relationship between total air volume within the lungs and their dielectric properties in vivo.

## 1. Introduction

The dielectric properties of biological tissues reveal the interaction mechanisms of tissue microstructure and electromagnetic fields relating to physiological and pathological conditions, and thus are of great importance in biophysical research and clinical applications [1]. Currently, the study of the dielectric properties of biological tissues has become popular in bioelectromagnetics [2] and bioelectrical impedance imaging [3] research because of its potential and substantial role in revealing the deep-rooted mechanism of life phenomena, optimizing and innovating clinical diagnostic methods, and actively promoting the innovative development of related medical devices.

In the field of research on dielectric properties of biological tissues, lung tissue is recognized as the most challenging research object due to its anatomical structure and physiological functions. Its complex structure includes a microscopic bronchial tree, numerous alveoli, a rich vascular network, and a lymphatic system [4], which together determine its dielectric properties. In addition, the dielectric properties of lung tissue are highly dynamic, changing with the expansion and contraction of alveoli, air exchange, and blood perfusion during the respiratory cycle. In particular, fluctuations in the air content in the alveoli can have a profound effect on the overall dielectric properties of lung tissue [5]; i.e., when the alveoli are filled with more air, the overall electrical resistance properties of the lung tissue are significantly altered due to the fact that air is a poor conductor. This phenomenon essentially forms the biophysical basis for the current widely used clinical technique of thoracic electrical impedance tomography (EIT), which provides a biophysical basis for assessing lung ventilation and fluid distribution by measuring changes in tissue electrical impedance [6]. Therefore, measuring lung tissue in vivo with the changing air content is key to—and a major challenge in—achieving accurate measurements of the dielectric properties of lung tissue.

In the last few decades, multiple research efforts have explored the dielectric properties of lung tissue. The research focus has been mainly on two aspects: on the one hand, the changes in the dielectric properties of lung tissues in pathological states have been analyzed in an attempt to reveal the alterations in the dielectric properties of tissues in different lung disease states and their correlation with the disease process. Sun et al. found that the permittivity and conductivity of lung metastatic lymph nodes were higher than those of normal lymph nodes in the frequency range of 1 MHz–4 GHz [7]. Wang et al. found significant differences in dielectric properties between human lung cancer tissues and normal tissues [8]. On the other hand, the dielectric properties of lung tissues under normal physiological conditions have also been investigated. Sebek et al. measured the dielectric properties of isolated porcine and bovine lung tissues in the frequency band of 500 MHz–6 GHz [9]. Colebeck et al. investigated the temperature dependence of the dielectric properties of isolated porcine lungs in the 500 MHz to 20 GHz band [10]. Nopp et al. characterized the air content of the alveoli in the frequency range of 100 Hz–100 MHz by means of an ex vivo inflation factor F determined by the sample mass and volume [11]. However, the lung tissue for these studies was directly stripped from the organism, and under in vitro experimental conditions, key factors such as air exchange could not be realistically represented due to the loss of support from the original physiological environment. Such a stripped state means that experimental results may not accurately reflect the dielectric properties of lung tissue under natural physiological conditions, thus becoming the biggest shortcoming of ex vivo experimental methods. As a result, current research mainly lacks effective noninvasive in vivo measuring techniques [12], and few studies have explored the relationship between lung tissue dielectric properties and air content in vivo.

At present, measurements targeting the dielectric properties of lung tissue cover both low- and high-frequency band techniques. Impedance methods are predominantly utilized for measurements in the low-frequency band, which is generally below 1 megahertz. For example, Witsoe et al. used the four-electrode method to measure the dielectric properties of lung tissue from the surface of a dog at a frequency of 100 kHz [13], but their method causes tissue damage and airtightness disruption, resulting in an inevitable reduction in measurement accuracy, and stray capacitance and inductance within the measurement system increasingly affect the results as the measurement frequency increases. In addition, the main high-frequency band measurement methods include the waveguide transmission method [14,15,16], coaxial transmission reflection method [17,18,19], and resonance measurement method [20,21,22]. For example, Vidjak et al. found an air content threshold in isolated sheep lungs using the open-ended coaxial probe technique in the range of 500 MHz to 8 GHz; below this limit, the dielectric properties of the lungs will change drastically with changes in air density [23]. However, their study is still limited by the fact that it is based only on simulation experiments in an ex vivo environment and not on observations under real physiological conditions in living animals. This indicates that although the study reveals the relationship between the dielectric properties of lung tissue in the ex vivo state and air content, additional consideration of the effects of more physiological factors may be required when interpreting the actual situation in vivo. The open-ended coaxial probe technique is a well-established method in transmission line measurements and is especially effective for high-frequency applications. This technique is noninvasive, meaning it can measure without damaging the sample, and it is not limited by the geometrical structure of the sample. Additionally, it enables continuous and accurate real-time monitoring of lung tissues in vivo across a broad frequency range. These features are essential for accurately capturing the dynamic changes in the dielectric properties of lung tissues as they respond to variations in air content during ventilation.

Furthermore, most previous studies have focused on the modeling of solid tissues, and these models usually assume that tissues have a relatively homogeneous material composition and that their dielectric properties are mainly influenced by the inherent dielectric properties of the tissue itself. However, the lung, as a tissue containing a large volume of air, has dielectric properties that change dynamically as the volume of gas changes during respiration. Therefore, there may be some uncertainty in the applicability and accuracy of modeling methods applicable to solid tissues when applied directly to the lungs. Considering the unique structure of lung tissue and the fluctuation in air content during respiration, existing modeling methods may need to be adapted and improved accordingly.

Prior to this, our research group’s work focused on designing and fabricating specialized probes, establishing measurement and calculation methods, and completing preliminary in vivo measurements to obtain data on the dielectric properties of rabbit lung tissue [24]. In this study, we aim to (1) investigate the relationship between in vivo dielectric properties of lung tissue and tidal volume based on rabbits and (2) conduct novel and accurate modeling and fitting analysis of the dielectric properties of lung tissue in vivo, with the goal of revealing the dielectric response of lung tissue at various tidal volume levels. As the first step, to explore the interaction between the in vivo dielectric properties of lung tissue and total air content over a wide frequency range, the open-ended coaxial probe was primarily employed in this study to focus on measurements in the high-frequency range (100 MHz–1 GHz). This study adopts the in vivo measurement technique, which does not cause damage to the lung tissue and, at the same time, precisely regulates the tidal volume inside the lungs, aiming to make the experimental conditions as close as possible to the actual physiological state so as to obtain more accurate and reliable data on dielectric properties.

## 2. Methods

### 2.1. Measurement System and Accuracy Verification

#### 2.1.1. Setup of the Measurement System

**Figure 1:**
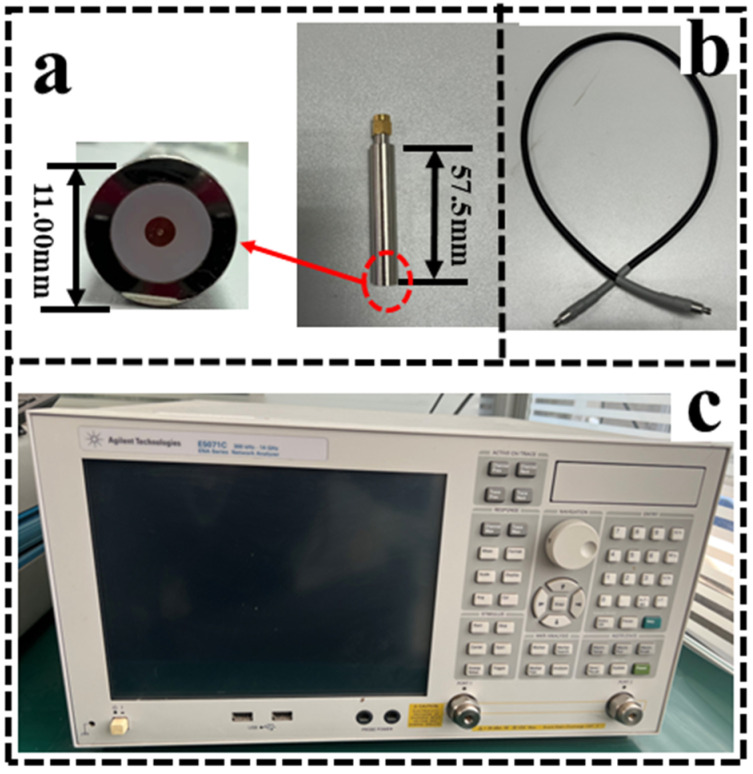
Experimental system and equipment. (**a**) The Teflon-filled probe was chosen to prevent errors caused by the penetration of tissue fluids into the probe during measurements, thus ensuring accurate measurements of the dielectric properties of tissue. (**b**) A coaxial cable for connection to the vector network analyzer. (**c**) The measurement band of the vector network analyzer: 100 MHz~1 GHz.

As shown in Figure 1, the entire experimental system used a custom-made Teflon-filled coaxial probe (length: 57.5 cm, diameter: 11.00 cm, permittivity: 2.02) with a mean error of no more than 2% [25], which was connected to a vector network analyzer (E5071C, Agilent Inc., Santa Clara, CA, USA) via a coaxial cable.

#### 2.1.2. Methods of System Accuracy Verification

The open-ended coaxial probe, as a proven methodology, has been extensively validated in terms of measurement means, computational models, and characterization (fitting) models [26]. The aim of this step is to validate the accuracy and reliability of the actual measurement steps and modeling process. Specifically, the validation will be divided into the following two sections:

(1) The accuracy of the measurement system is verified using a standard sample of NaCl (sodium chloride) solution with known properties, which provides a reliable reference for calibrating the measurement equipment due to its stable conductivity and permittivity at different concentrations [27]. By comparing experimental data with theoretical values, the accuracy of the measurement system can be evaluated. Since the closer the calibration solution is to the dielectric properties of the tissue, the better the calibration effect is, three NaCl solutions with concentrations of 0.001, 0.0015, and 0.0008 mol/L were selected for the measurement.
(2) Ex vivo porcine liver was selected to verify the reliability of the measurement system. This is because porcine liver tissue has stable dielectric properties and is often used as a reference standard in biological tissue studies [28], and its tissue structure and physiological properties are different from those of lung tissue, so that the influence of the air on the measurement results can be excluded. The same experimental set-up and procedures were maintained, and 10 replicates of the experiment were performed using isolated porcine liver tissue.

### 2.2. The Procedures of Rabbit Lung Measurement

**Figure 2:**
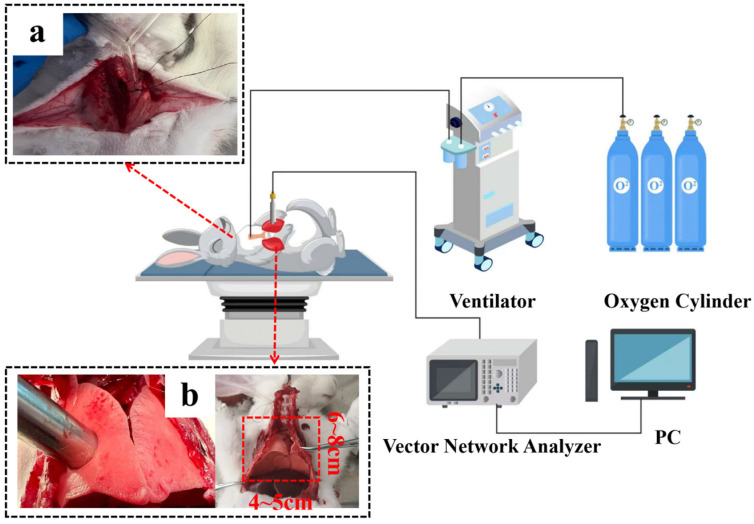
Experimental diagram of lung tissue measurements in rabbits. (**a**) Tracheal intubation was used to effectively maintain the rabbits’ respiratory patency, as well as to reduce the potential effects caused by airway obstruction, thus improving the accuracy and reliability of the measurement data. (**b**) The size of the lung tissue being measured is approximately 4 to 5 cm in length and 6 to 8 cm in width.

Twenty-five adult male rabbits were used in the experiment. The schematic diagram of the experiment is shown in Figure 2. At the beginning of the experiment, the rabbits were mechanically ventilated using volume-controlled ventilation (VCV) mode, and the ventilation parameters were set to include a tidal volume (VT) of 50 mL and an inspiratory/expiratory ratio (I:E) of 1:2. After confirming that the rabbits had reached a deep anesthesia state, the rabbits were tracheally intubated, and the airway patency was carefully examined. Next, the skin was cut transversely along the raphe of the rabbit’s sternum, and then the ribs were cut with scissors about 3 cm on either side of the sternum, the sternum was gently lifted, and the lower part of the sternum was carefully peeled off, taking care to avoid damaging blood vessels. Subsequently, the anterior wall of the thorax was thoroughly removed to fully expose the left and right lung tissues. To ensure that stable measurement data were obtained, the rabbits were kept at a constant temperature of 38.5–39.5 degrees Celsius with thermal blankets during the experiment. According to the actual physiological limitations of the rabbit’s lungs, six different tidal volumes were set up, namely 30 mL, 40 mL, 50 mL, 60 mL, 70 mL, and 80 mL. Measurements were taken in order of tidal volume from smallest to largest, with 5 min intervals for each group and 10 min elution intervals.

Before taking measurements, we gently rinsed the surface of the sample with saline solution to remove any blood residue that might have been present. To ensure close contact, without air gaps, between the probe and the target tissue, a thin layer of matching medium (e.g., 0.9% saline) was applied to the probe contact surface. This medium has dielectric properties similar to those of tissue fluid and helps to improve coupling efficiency. When placing the probe, gentle and even pressure was used to ensure that the probe adhered smoothly to the surface of the lung tissue while avoiding excessive pressure that could have damaged the structure of the lung tissue.

After the rabbit had completed full inspiration, the air intake port was closed temporarily to ensure that the lungs remained stable at the end of the inspiratory phase for at least 1 min, and at this time, the dielectric properties of the rabbit’s lung tissues were measured. To ensure the accuracy and reliability of the measurement results, measures such as fixing the position of the rabbit and recording the coordinates of the measurement points in the right lobe of the lungs were taken to minimize the errors introduced by changes in the measurement position, and the measurements were repeated three times to obtain the average value.

### 2.3. Data Analysis

#### 2.3.1. Cole–Cole Model

The expression for the complex permittivity *ε_r_*, which usually describes the dielectric properties of the tissue, is shown in Equations (1) and (2) [29]:

(1) $$\begin{array}{c} \varepsilon_{r}^{*}\left(\omega \right)=\varepsilon_{r}^{\prime }+{j\varepsilon }_{r}^{\prime \prime } \end{array}$$

(2) $$\varepsilon_{r}^{\prime \prime }=-\frac{\sigma_{s}}{\omega \varepsilon_{0}}$$

where *ε_r_*′ is the real part permittivity of the tissue, commonly referred to as the dielectric constant; *ε*_0_ denotes the permittivity of vacuum; *ω* denotes the angular frequency; *ε_r_*″ is the imaginary part of the permittivity; *j* denotes the imaginary unit; and *σ_s_* is the static conductivity.

The dielectric properties of biomaterials over a wide frequency range can be expressed by the Cole–Cole model. In this work, measurements were performed in the 100 MHz–1 GHz band. Therefore, when searching for a Cole–Cole fit model, it was expected that the number of poles would be less than 4 [7]. To simplify the model parameters, a first-order model was employed for fitting, as shown in Equation (3) [30]:

(3) $$\begin{array}{c} \varepsilon_{r}^{*}\left(\omega \right)=\varepsilon_{\infty }+\frac{\Delta \varepsilon }{1+{\left(j\omega \tau \right)}^{1-\alpha }}+\frac{\sigma_{s}}{j\omega \varepsilon_{0}} \end{array}$$

where *ε*_∞_ is assigned values between 2.4 and 4.2 depending on the water content of the tissue, ∆*ε* represents the changes in the permittivity of the dispersion, *τ* denotes the time constants of the dispersion, *α* is the relaxation factor, and *σ_s_* is the static conductivity.

#### 2.3.2. An Improved Cole–Cole Model Considering Air Content

In the original Cole–Cole model, the complex permittivity is usually expressed as a complex number containing a real part (related to capacitive behavior) and an imaginary part (related to energy loss or dielectric loss). In the initial phase of our study, we found that the Cole–Cole model did not provide a satisfactory fit for the in vivo measurement data. The presence of air within the lungs appears to be a significant contributing factor to this mismatch. Therefore, we considered developing an improved model to better account for these effects and enhance the accuracy of the measurements. Based on the Cole–Cole model, in order to more accurately model and describe the dielectric properties of lung tissue at different frequencies, this study proposes to set the dispersive permittivity and static conductivity as linear frequency-dependent functions. As shown in Equation (4), in the improved model, linear frequency-dependent functional coefficient A was introduced to provide more flexibility in modeling the complex nature of the frequency-dependent changes in the dielectric properties of the lung tissue, especially considering that changes in the air composition within the lung tissue can lead to significant variations in the dielectric properties under different states of ventilation:

(4) $$\varepsilon_{r}^{*}\left(\omega \right)=\varepsilon_{\infty }+\frac{A\omega +\Delta \varepsilon }{1+{\left(j\omega \tau \right)}^{1-\alpha }}+\frac{\sigma_{s}}{j\omega \varepsilon_{0}}$$

where *A* denotes the first-order linear coefficient associated with the frequency-dependent term of the initial permittivity change Δ*ԑ*.

#### 2.3.3. Fitting Effect of the Models

In this experiment, the measured data were fitted to a dielectric parameter model using the simulated annealing (SA) algorithm. The algorithm was controlled and improved by a cooling schedule to improve the fit. A measure of the overall accuracy of the fit was calculated from the average fitting error $\bar{E_{rr}}$ as shown in Equation (5):

(5) $$\begin{array}{c} \bar{E_{rr}}=\frac{\sum_{i=1}^{N}\sqrt{{\left(\frac{Re(\varepsilon_{1}(\omega ))-Re(\varepsilon_{2}(\omega ))}{Re(\varepsilon_{2}(\omega ))}\right)}^{2}+{\left(\frac{Im{(\varepsilon }_{1}(\omega ))-Im{(\varepsilon }_{2}(\omega ))}{Im(\varepsilon_{2}(\omega ))}\right)}^{2}}}{N}\times 100\% \end{array}$$

where *Re*(*ε*_1_(*ω*)) and *Im*(*ε*_1_(*ω*)) are the real or imaginary parts of the measured complex permittivity. *Re*(*ε*_2_(*ω*)) and *Im*(*ε*_2_(*ω*)) are the real or imaginary parts of the calculated complex permittivity (obtained by fitting).

The fit curve *R*^2^ is a statistical indicator of the degree of match between the actual measurements and the fitted curve. Its value ranges from 0 to 1. The closer the value is to 1, the higher the degree of fit of the fitted curve to the actual measurements, indicating that the fitted model is more reliable.

(6) $$R^{2}\equiv 1-\frac{SS_{res}}{SS_{tot}}$$

In the above equation, Equation (6), *SS_res_* denotes the sum of the squares of the differences between the measured and calculated values, i.e., the error between the measured and calculated values, and *SS_tot_* denotes the squared difference, which represents the degree of dispersion of the measured values. The larger this is, the more dispersed it is.

#### 2.3.4. Statistical Analysis

In statistics, a normality test was first executed on the collected data to determine whether the distribution pattern conformed to the law of normal distribution. If the data were found to be normally distributed, the mean and standard deviation were used to describe the tendency of the data to concentrate and the degree of variability, and parametric statistical methods, such as *t*-tests, were used to compare whether there was a significant difference between the means of the dielectric properties of the lung tissues at different tidal volumes. However, if the data did not satisfy normal distribution, the median was used instead of the mean at this point to characterize the concentration trend of the data, in combination with other non-parametric statistics (e.g., quartiles, box plots, etc.) to reflect the variability of the data. In order to assess whether there was a significant difference in the dielectric properties of rabbit lung tissue at the six different tidal volumes, the KruskalWallis (K-W) test was used with the significance level set at 0.05 (*p* < 0.05). The K-W test is a non-parametric method for comparing the location of more than two independent sample distributions. It is suitable for data sets that do not meet the assumptions of analysis of variance (ANOVA), especially when the data do not conform to a normal distribution, or there are outliers. Given that dielectric properties are closely related to electromagnetic wave frequencies, this study focused on two specific frequency points, 433 MHz and 915 MHz, which are widely used in the industrial, scientific, and medical (ISM) bands and have important applications in the development of medical devices such as radiofrequency and microwave ablation devices [7].

## 3. Results

### 3.1. Results of System Accuracy Verification

#### 3.1.1. Measurement of Standard Solutions

**Figure 3:**
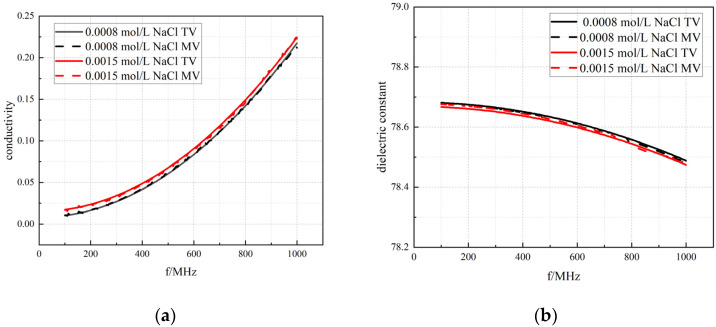
The NaCl solution of (**a**) conductivity and the (**b**) dielectric constant. Black solid line: 0.0008 mol/L NaCl solution theoretical value; black dotted line: 0.0008 mol/L NaCl solution measured value; red solid line: 0.0015 mol/L NaCl solution theoretical value, red dotted line: 0.0015 mol/L NaCl solution measured value.

Firstly, in this study, 0.001 mol/L NaCl solution was selected as the calibration reference standard to fine calibrate. Repeated measurements were carried out for NaCl solutions with concentrations of 0.0015 mol/L and 0.0008 mol/L, respectively, at a room temperature of 24 degrees centigrade. Figure 3 illustrates the dielectric constant and conductivity of NaCl solutions obtained through experimental measurements, compared against theoretical values from two authoritative scientific papers [27,29], which are widely recognized in the field. The experimental results show that the measured parameters of the dielectric properties of various concentrations of NaCl solutions demonstrate a high degree of consistency with the theoretical predictions, and all the errors are strictly controlled within the threshold value of ±5%, which verifies the reliability of the measurement system used in this study.

#### 3.1.2. Measurement of Ex Vivo Porcine Liver

**Figure 4:**
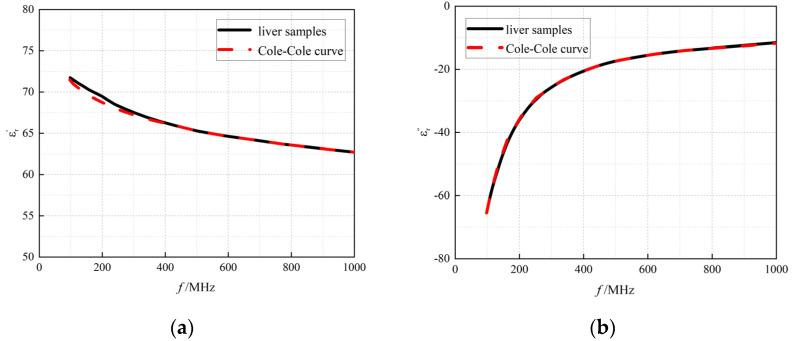
Cole–Cole fitted curves of (**a**) the real part of the complex permittivity and (**b**) the imaginary part of the complex permittivity as a function of frequency. Black solid line: measured data of isolated liver; red dashed line: fitted data of isolated liver.

In the experimental procedure, the dielectric properties of porcine liver tissue were measured using an open-ended coaxial probe in the specified frequency range (100 MHz to 1 GHz), and the data were fitted using the Cole–Cole model. As shown in Figure 4, the results demonstrated that the average error $\bar{E_{rr}}$ in fitting the dielectric properties of isolated porcine liver tissue by the Cole–Cole model was within 2%, and the coefficients of determination of the real and imaginary parts of the complex permittivity R^2^ exceeded 0.90. Thus, the reliability of the measurement system and the Cole–Cole model were verified for liver tissue.

### 3.2. Dielectric Properties of Lung Tissue with Changing Air Volume

**Figure 5:**
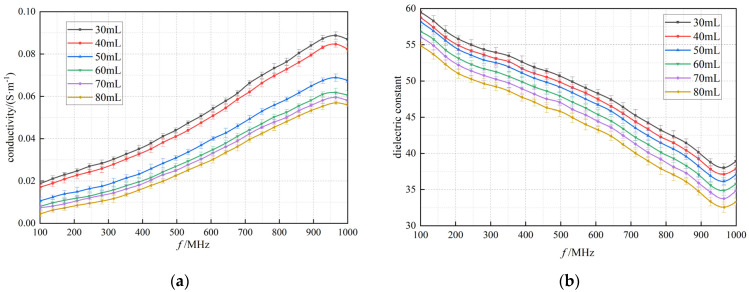

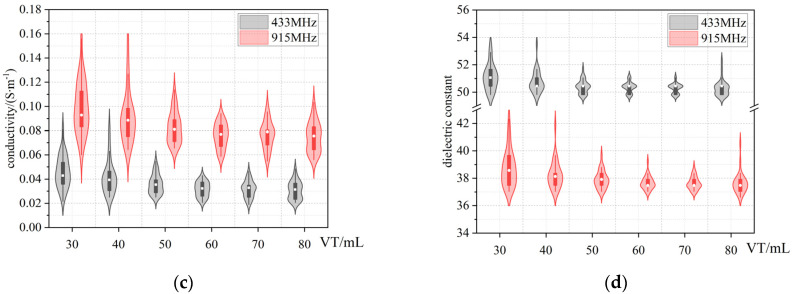
In measurements of the left lung lobe of the rabbits, each measurement point was repeated three times. Experimental results on the variation in (**a**,**c**) conductivity and (**b**,**d**) the dielectric constant with 6 sets of tidal volumes (30 mL, 40 mL, 50 mL, 60 mL, 70 mL, and 80 mL, respectively). Black box: data distribution of the dielectric properties of lung tissue measured at 433 MHz. Red box: data distribution of the dielectric properties of lung tissue measured at 915 MHz.

In this experimental study, the dielectric properties of rabbit lung tissue were systematically measured by gradually applying six different levels of tidal volume, aiming to simulate and investigate the changes in the dielectric properties of the lung tissue under different inflation levels. As shown in Figure 5, the results showed that the electrical conductivity and permittivity of the rabbit lung tissue showed a clear trend of decreasing with a gradual increase in tidal volume over the range of frequency bands tested. This finding is in agreement with previous findings by Nopp et al. on the dynamic relationship between air content within lung tissue and its dielectric properties [5], suggesting that the dielectric properties of lung tissue do indeed change regularly with the volume of air within the lung [24]. Further analysis of the data at two representative frequency points (433 MHz and 915 MHz) using the K-W test showed the same trend in conductivity and dielectric constant, and the differences were statistically significant (*p* < 0.01) for the six groups at different tidal volumes.

### 3.3. Cole−Cole Model Fitting

**Figure 6:**
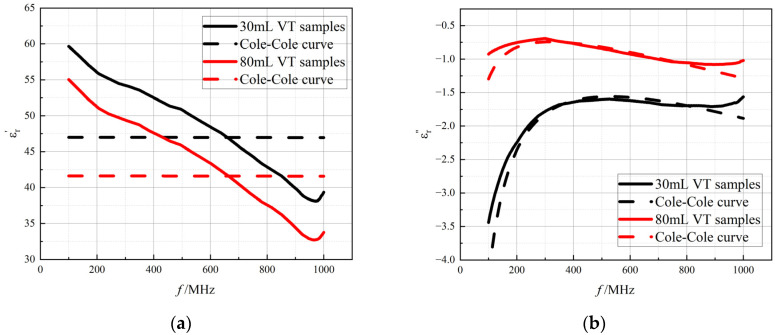
The Cole–Cole model fitted curves of (**a**) the real part of the complex permittivity and (**b**) the imaginary part of the complex permittivity as a function of frequency. Red solid line: measured data for tidal volume of 80 mL lungs; red dashed line: fitted data for tidal volume of 80 mL lungs; black solid line: measured data for tidal volume of 30 mL lungs; black dashed line: fitted data for tidal volume of 30 mL lungs.

In this study, the experimentally obtained data on the dielectric properties of rabbit lung tissue were fitted and analyzed using the Cole–Cole model, and the complex permittivity obtained from actual measurements was compared and validated against the model predictions. In order to visualize the process of changes in the dielectric properties of lung tissue with air content, changes in tidal volume were used as a means of simulation to reflect the dielectric behavior of the lungs under different inflation states. Figure 6 illustrates two extreme tidal volume conditions: on the one hand, it indicates a relatively high intra-alveolar air content at a maximum tidal volume of 80 mL when the lung tissue is in a fully inspiratory state; on the other hand, it indicates a low intra-alveolar air content corresponding to a minimum tidal volume of 30 mL near the end of expiration. According to Table 1, the average fitting error $\bar{E_{rr}}$ for lung tissue using the Cole–Cole model exceeded 10%, indicating that the model is biased in describing the in vivo dielectric properties of lung tissue with different tidal volumes. According to the definition of the coefficient of determination, the fit of the imaginary part was significantly better than that of the real part, but it still failed to reach the ideal state. The model needs to be further improved to describe the complex dielectric behavior of lung tissue.

### 3.4. The Improved Cole–Cole Model Considering Air Content

**Figure 7:**
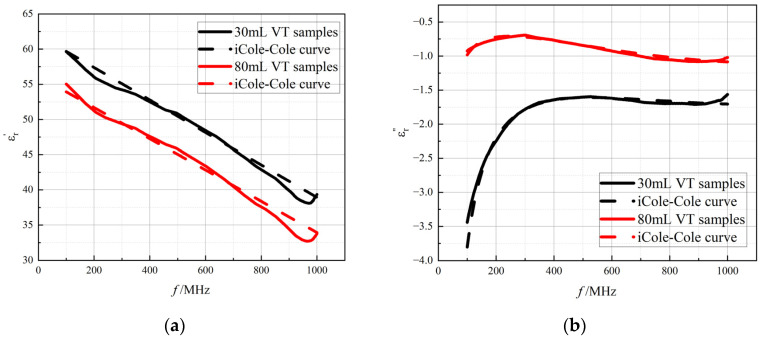
The improved Cole–Cole model fitted curves of (**a**) the real part of the complex permittivity and (**b**) the imaginary part of the complex permittivity as a function of frequency. Red solid line: measured data for tidal volume of 80 mL lungs; red dashed line: fitted data for tidal volume of 80 mL lungs; black solid line: measured data for tidal volume of 30 mL lungs; black dashed line: fitted data for tidal volume of 30 mL lungs.

In the present study, the investigation of the dielectric properties of rabbit lung tissue reveals the limitations of the Cole–Cole model in fitting its measured data, showing a certain degree of inconsistency between model fitting and actual tissue properties. Considering the complexity of the microstructure of biological tissues, as well as the possible frequency-dependent effects of intrinsic moisture distribution, ionic concentration, and the distribution of inhomogeneous media such as air in the tissues, the existing models may not be able to adequately describe the dynamics of the dielectric parameters of the lung tissues as a function of frequency. To this end, we transformed the permittivity of the dispersion (∆*ε*) parameter, which was originally fixed in the Cole–Cole model, into linear frequency-dependent functional expressions. In the experiment, the good performance of this modified model was verified (see Figure 7). Model parameter A is designed to be small since they are multiplied by frequency values in the formula. In the comparative results in Table 2, the improved model made significant progress in fitting the dielectric properties of lung tissue. Compared to the Cole–Cole model, the improved model significantly reduces the fitting error $\bar{E_{rr}}$ to no more than 5%, implying that the improved model is able to more accurately describe and fit the changes in the dielectric properties of the lung tissue in different states (see Table 3). The dielectric parameters of in vivo lung tissue at different tidal volumes detailed in Table 3 reflect the dynamic response of the dielectric properties of lung tissue to changes in tidal volume, which provides a solid foundation for subsequent data analysis and mining work. In our study, the experimental results show that the values of α are close to zero and the Cole–Cole model degenerates to the classical Debye model. The actual measured values of the lung tissue dielectric properties at the two extreme tidal volume conditions are shown in comparison with the fitted curves of the improved model, which further confirms that the improved model has a higher fitting efficacy and practical value in dealing with the variation in the lung tissue dielectric properties under different respiratory states. The Cole–Cole model is incapable of calculating the dielectric properties of lung tissues under different tidal volumes, and the improved model can accurately fit the dielectric properties of lung tissue under different tidal volume conditions; thus, the improved model can more accurately fit the variation patterns of the complex permittivity (including the real part of *ε_r_*′ and the imaginary part of *ε_r_*″) under various tidal volume states.

## 4. Discussion

In this study, the non-destructive in vivo measurement technique—using an open-ended coaxial probe—was employed to investigate the dielectric properties of in vivo lung tissue at different tidal volumes in the high-frequency range of 100 MHz to 1 GHz. By accurately regulating the tidal volume inside the lungs, the experimental conditions were ensured to simulate the real physiological state as much as possible so as to obtain more accurate and reliable data on the in vivo dielectric properties of lung tissues. Furthermore, a novel fitting model was constructed to reflect the dielectric properties of in vivo lung tissue under a physiological state. In the new model, the smaller values of static conductivity *σ*_s_ may be due to the fact that air filling significantly alters the dielectric properties of lung tissue during lung ventilation. The model parameter *α*, which represents the degree of distribution of different relaxation times in the mixture, was found to have values equal to zero in the finite frequency interval from 100 MHz to 1 GHz, which may be attributed to the fact that the main determinants of the dielectric properties of the lung tissues in this frequency range come from the polarization and relaxation effects of the water molecules due, in particular, to counterion polarization and Maxwell–Wagner effects [31].

Moreover, we found that the permittivity of lung tissue at any given tidal volume can be calculated as a weighted average of the permittivity of the tissue itself and the permittivity of air. The trend in the permittivity of lung tissue is primarily driven by the dilution effect of air within the tissue. This relationship is further corroborated by our experimental data, presented as dispersion curves, which reveal systematic and regular changes in the dielectric properties of lung tissue as tidal volume increases. While our current study provides initial evidence of this relationship, precisely quantifying the weighting factors requires additional experimental data and more rigorous theoretical analysis.

**Figure 8:**
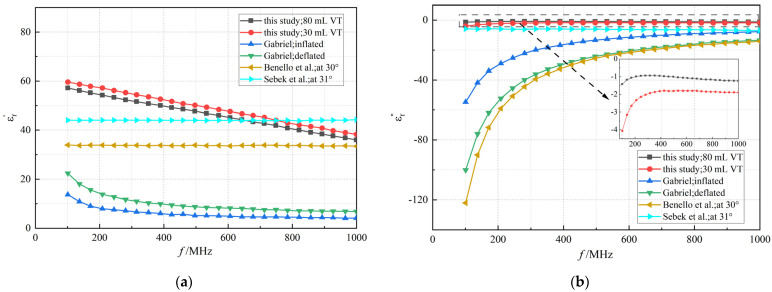
All lung dielectric properties references (Gabriel 1996, [32,33,34]; Benello 2018, [35]; Sebek 2019, [9]). The comparison of real (**a**) and imaginary (**b**) parts of the complex permittivities.

Previous research on lung tissue’s dielectric properties has often used isolated samples or indirect measurement methods [9,32,33,34,35], which do not adequately reflect the tissue’s dynamics under actual physiological conditions. This study contrasts earlier ex vivo data with current in vivo measurements, revealing significant discrepancies, particularly in simulating dynamic changes during respiration. As shown in Figure 8, the real component of the complex permittivity measured in vivo was higher, and the imaginary component was lower compared to past studies. This difference might be attributed to the physiological activities of the lung during ventilation, affecting dielectric properties through variations in air content. When performing in vivo measurements, the imaginary part of the dielectric properties of lung tissue does not exhibit a complete absence of dispersion but, instead, a rather weak dispersion effect. The disparity between real and imaginary values underscores a fundamental difference in the electromagnetic behavior of lung tissue in vivo versus ex vivo.

The present study only initially investigated the dielectric properties of normal lung tissues, and subsequent studies should explore the changes in the dielectric properties of pathological lung tissues (e.g., lung nodules, pneumonia, lung cancer, etc.); this exploration is important for understanding the mechanisms of disease development, early diagnosis, and assessment of treatment effects. Some pathological states, such as lung solidification due to pneumonia, may severely affect the air content in the alveoli, thereby altering the dielectric properties of the tissue. By comparing the dielectric properties of normal lung tissue with those of lung tissue in various pathological states, it is possible to identify biomarkers for specific diseases, which can help develop new noninvasive or minimally invasive diagnostic techniques. In addition, understanding the pattern of change in the dielectric properties of these pathological tissues can also help guide and optimize the techniques and methods of using electromagnetic waves (e.g., radiofrequency, microwave, etc.) to treat lung diseases.

The primary limitation of this study is that it only considers the relationship between tidal volume and dielectric properties. Since measuring the functional residual capacity or total lung capacity of rabbits requires placing them in a sealed environment and using methods such as body plethysmography or helium dilution, it was not feasible to measure these parameters alongside their dielectric properties due to the limited conditions. Furthermore, to minimize the variation in lung expansion at the same tidal volumes, we selected rabbits with similar features (gender, age, body weight), allowing us to assume that the functional residual capacity or total lung capacity was essentially consistent, or that the lung expansion at the same tidal volumes was similar, thus ensuring the rationality of our measurement data. In future research, a possible solution might be to determine the functional residual capacity or total lung capacity using body plethysmography or helium dilution methods before intubating the rabbits. By assuming these values remain constant in subsequent experiments, it would be possible to establish the relationship between the total air in the lungs and dielectric properties.

The anatomy of rabbit lungs is similar to, but also different from, that of human lungs, which together affect their applicability and limitations in scientific research [36]. First, the rabbit is a common laboratory animal, which makes it suitable for the initial stages of research to confirm the reliability of the methods used and lay the foundations for subsequent research. Furthermore, the average diameter of the alveoli in the rabbit lung is slightly smaller than that in the human lung, but this difference is likely to have a minor effect on the dielectric properties, which are influenced more by the composition of the tissue than by the absolute size. Secondly, the rabbit lung is divided into two left lobes and four right lobes, while the human lung is divided into two left lobes and three right lobes. The ratio between the upper and lower lobes of the rabbit lung is different from that of the human lung, which may affect the direct applicability of the results of some specific types of research, especially regarding lobe-specific pathological changes. However, overall, the impact of this difference may be reduced if research focuses on the combined dielectric properties of the entire lung rather than specific lung lobes. Finally, the normal body temperature of rabbits ranges from 38.5 to 39.5 degrees Celsius, which is higher than the average human body temperature (approximately 36.5 to 37.5 degrees Celsius). Higher body temperature affects physiological states such as the metabolic rate, water content, and blood flow of biological tissues, which may, in turn, affect the dielectric properties of tissues. In summary, although rabbit lungs are similar to human lungs in many ways and are suitable for basic research, anatomical differences (e.g., lobular structure) and physiological parameters (e.g., body temperature) should be considered carefully when directly applying research results to humans.

## 5. Conclusions

In this study, we explored the dielectric properties of in vivo rabbits’ lung tissues at various tidal volumes using an open-ended coaxial probe within the 100 MHz to 1 GHz range. By measuring in real physiological respiratory states, we obtained reliable data showing how tidal volume changes affect lung tissue dielectric properties. An improved Cole–Cole model was introduced, effectively fitting these relationships.

We hypothesized trends in lung tissue permittivity with tidal volume and frequency dispersion effects. Experimental results confirmed these hypotheses, demonstrating the improved Cole–Cole model’s effectiveness across different tidal volumes. Selecting rabbits with similar characteristics minimized variability, ensuring measurement consistency.

Our methodology offers significant advantages: noninvasive in vivo measurements, an improved fitting model for lung tissue, and insights into air volume impacts on dielectric properties. However, limitations include focusing solely on tidal volume without considering other lung volumes like functional residual capacity.

Future research should examine lung tissue dielectric property changes under diseases like pneumonia or lung cancer, crucial for understanding disease mechanisms and treatment assessment. Utilizing larger animals with lung physiology closer to humans can aid in simulating human gas exchange processes. Additionally, incorporating high- and low-frequency data could help construct comprehensive lung tissue dielectric maps for more accurate physiological condition simulations.

## Figures and Tables

**Table 1:** The Cole–Cole model fitted curves of lung tissue.

VT (mL)	$\bar{E_{rr}}$	$R_{1}^{2}$ (Real Part)	$R_{2}^{2}$ (Imaginary Part)
30	12.85%	0.084	0.801
40	13.78%	0.084	0.631
50	16.73%	0.079	0.302
60	15.10%	0.062	0.398
70	16.31%	0.083	0.720
80	15.57%	0.107	0.491

**Table 2:** The improved Cole–Cole model fitting curves of lung tissue.

VT (mL)	$\bar{E_{rr}}$	$R_{1}^{2}$ (Real Part)	$R_{2}^{2}$ (Imaginary Part)
30	2.16%	0.983	0.976
40	2.95%	0.989	0.974
50	2.18%	0.984	0.948
60	2.87%	0.988	0.932
70	3.18%	0.976	0.932
80	3.18%	0.982	0.963

**Table 3:** The improved Cole–Cole model result of lung tissue fitting parameters.

	VT (mL)	30	40	50	60	70	80
Parameter							
*ε*_∞_		3.48	3.22	3.63	2.28	3.81	2.13
∆*ε*		58.52	57.81	58.59	56.43	55.07	54.01
Τ (ps)		6.04	5.91	5.15	4.93	5.31	5.02
*α*		0	0	0	0	0	0
*σ_s_* (×10^−3^ S/m)		19.96	17.56	10.48	7.90	6.61	4.55
A (×10^−9^)		−3.66	−3.74	−3.74	−3.68	−3.82	−3.53

## Data Availability

The original contributions presented in this study are included in the article; further inquiries can be directed to the corresponding author.
